# A Polyamidoamine Dendrimer-Based Electrochemical Immunosensor for Label-Free Determination of Epithelial Cell Adhesion Molecule- Expressing Cancer Cells

**DOI:** 10.3390/s19081879

**Published:** 2019-04-19

**Authors:** Jianguo Xu, Xinxin Wang, Chao Yan, Wei Chen

**Affiliations:** School of Food and Biological Engineering, Engineering Research Center of Bio-Process, MOE, Hefei University of Technology, Hefei 230009, China; wxx251321@163.com (X.W.); yanchao19930215@foxmail.com (C.Y.)

**Keywords:** label-free, immunosensor, polyamidoamine dendrimer, epithelial cell adhesion molecule (EpCAM), electrochemical impedance spectroscopy (EIS)

## Abstract

A new electrochemical immunosensor for cancer cell detection based on a specific interaction between the metastasis-related antigen of epithelial cell adhesion molecule (EpCAM) on the cell membrane and its monoclonal antibody (Anti-EpCAM) immobilized on a gold electrode has been developed. The amino-terminated polyamidoamine dendrimer (G6 PAMAM) was first covalently attached to the 3-mercaptopropionic acid (MPA)-functionalized gold electrode to obtain a thin film, and then completely carboxylated by succinic anhydride (SA). Next, the Anti-EpCAM was covalently bound with the G6 PAMAM to obtain a stable recognition layer. In the presence of the EpCAM expressing hepatocellular carcinomas cell line of HepG2, the specific immune recognition (Anti-EpCAM/EpCAM) led to an obvious change of the electron transfer ability. The properties of the layer-by-layer assembly process was examined by cyclic voltammetry (CV) and electrochemical impedance spectroscopy (EIS). The final determination of HepG2 cells was performed in the presence of the reversible [Fe(CN)_6_]^3−/4−^ redox couple using impedance technique. Based on the advantages of PAMAM nanomaterial and immune reaction, a linear response to HepG2 cells ranging from 1 × 10^4^ to 1 × 10^6^ cells mL^−1^ with a calculated detection limit of 2.1 × 10^3^ cells mL^−1^ was obtained. We expect this method can provide a potential tool for cancer cell monitoring and protein expression analysis.

## 1. Introduction

As is well known that cancer is considered as one of the most mortal diseases. The earlier we diagnose it, the less risk we face. Cancer biomarkers are molecules including DNA, RNA, proteins, and metabolites [1,2,3,4,5,6], which can indicate the presence of cancers and provide essential information about the cell signaling, migration, proliferation, and differentiation. Therefore, there is a continual demand for cancer biomarkers detection to meet the public demands for better disease diagnosis and therapy methods. Epithelial cell adhesion molecule (EpCAM), one of the transmembrane glycoproteins that is closely related with cancer metastasis [7], is highly expressed on many cancer cells [8]. It leads to the adhesion of circulating tumor cells (CTCs) to the vascular endothelial bed, which was strongly evidenced as the crucial starting point in the metastatic cascade and acts as the essential index to predict cancer metastasis progression and survival rates. EpCAM accounts for >90% of cancer mortality and represents an ideal biomarker candidate for cancer cell detection and monitoring. 

Up to now, several methodologies for cancer cell analysis have been built based on specific immune recognition between the monoclonal antibody of Anti-EpCAM and the EpCAM on cancer cell membranes. Flow cytometry [9,10,11], fluorescence microscopy [12] and microchip technology [13,14,15] are typical representatives with a certain degree of success. However, flow cytometry and fluorescence microscopy are limited by the need for labelled reagents and expensive instruments. Microchip technology is limited by the requirements of complex chip fabrication processes and skilled technicians. All these obstacles greatly inhibit their application for on-site and point-of-care tests. Most recently, particular attention has been paid to the application of electrochemical methods due to their intrinsic advantages of low cost, rapid response, easy miniaturization and compatibility with microfabrication technology [16]. Among them, electrochemical impedance spectroscopy (EIS) is the most effective and convenient analytical tool that requires no labeling of redox active moieties [17,18,19,20,21]. Via the useful EIS, the immobilization or bio-recognition events at the electrode surfaces result in the changes of the capacitance and interfacial electron transfer resistance, and can be well reflected by EIS value changes. Therefore, the EIS technique provides a powerful platform for immunosensor design and cell detection [22,23].

Dendrimers are a type of macromolecules with defined molecular weight, size, and three-dimensional steric structures with sufficient surface functional groups [24]. The applications of the nanoscale polyamidoamine (PAMAM) dendrimers combined with electrochemical approaches have been widely reported [25]. Deng et al. used ferrocenecarboxaldehyde, G4 PAMAM and a signal antibody to construct a trimer complex as a tracing tag for an electrochemical luminescence (ECL) immunoassay with a wide calibration range, excellent stability and acceptable reproducibility to detect carcinoembryonic antigen (CEA) [26]. Zhuo et al. have once designed a signal-on ECL immunosensor employing hollow gold nanospheres, PAMAM dendrimers and L-cysteine as the promoter for highly sensitive measurement of CEA [27]. These achievements verified that construction of electrochemical biosensors with dendrimers as linkers is an effective pathway to obtain desired assay performances, and more endeavor is encouraged to study and broaden its application fields. 

Inspired by this situation, in this work, we investigated the suitability of sixth generation PAMAM dendrimers (G6 PAMAM), a polyamide-amine type of dendritic polymers with 256 primary amine groups on the surface, for the development of a cancer cell-based immune EIS biosensor. Owing to the fact that hepatocellular carcinoma is the most common primary liver malignancy [28], we chose the HepG2 with positively-expressed EpCAM cell line as the target cells [9,29]. Our experimental results revealed that the cancer cells can be detected with good sensitivity and specificity without any complicated manipulations or labeling, indicating the method has practical potential for biomedical studies and disease diagnosis. Unlike flow cytometry, fluorescence microscopy and microfluidic chips, the detection of tumor cells can be carried out on the electrode surface and maybe further used for CTC detection since the identification of CTCs usually relies on the presence of EpCAM on tumor cell membranes [30].

## 2. Materials and Methods

### 2.1. Chemicals and Apparatus

The MPA was purchased from J&K Chemical Ltd (Beijing, China). G6 PAMAM was obtained from WeiHai CY Dendrimer Technology Co., Ltd. (Wei hai, Shandong, China) 1-ethyl-3-(3-dimethylaminopropyl) carbodiimide (EDC), N-Hydroxysuccinimide (NHS), and succinic anhydride (SA) were obtained from Aladdin Inc. (Shanghai, China). Bovine serum albumin (BSA) was from YuanHeng ShengMa Biology Technology Research Institute (Beijing, China). Anti-EpCAM and sialyl Lewis X (sLe^X^) were ordered from Abcam Ltd. (Hong Kong) and EMD Chemicals, Inc. (San Diego, CA, USA), respectively. Phosphate-buffered saline (PBS, pH 7.4) containing 136.9 mM NaCl, 2.7 mM KCl, 10.0 mM Na_2_HPO_4_ and 1.8 mM KH_2_PO_4_ was sterilized before use. Other reagents were of analytical grade. All aqueous solutions were prepared by ultrapure water (≥18 MΩ) purified with a Kertone Lab MINI system (Kertone Co., LTD, Changsha, China). Cyclic voltammetry (CV) and EIS experiments were performed with a CHI660E electrochemical workstation (CHI Instruments, Shanghai Chenhua Instruments Inc., Shanghai, China), and the three-electrode system was composed by a 3-mm-diameter gold working electrode, a Ag/AgCl reference electrode and a platinum (Pt) wire counterelectrode.

### 2.2. Pre-Preparation of Gold Electrode

Firstly, a gold electrode was polished carefully with alumina slurries (1, 0.3, 0.05 μm). After sonication in double-distilled water and ethanol solution for 30 s, the electrode was rinsed with double-distilled water and allowed to dry at room temperature. Then, it was electrochemically cleaned in 0.5 M H_2_SO_4_ by cyclic potential scanning between −0.2 and 1.6 V until a standard cyclic voltammogram was obtained. At last, the gold electrode was rinsed with copious amounts of double-distilled water and dried before use.

### 2.3. Modification of Gold Electrode

The PAMAM-modified electrode was prepared according to a previously described procedure [31]. Then the modified electrode was immersed into SA (167.0 μM) solution to completely carboxylate the amino groups [8]. After rinsing with double-distilled water to remove the excess reagents, the gold electrode was immersed in the mixture of EDC (20 mM) and NHS (40 mM) for 2 h to activate the carboxyls. This is followed by dropping 12 μL of Anti-EpCAM on the electrode surface to react for 8 h and form stable covalent bonds. Subsequently, the modified electrode was soaked in 1% BSA for 1 h to block the residual active sites. Finally, 12 μL of HepG2 cell suspension at concentrations ranging from 10^4^ cells mL^−1^ to 10^6^ cells mL^−1^ was deposited on the Anti-EpCAM-immobilized electrode and incubated at 37 °C for another 1 h. The already obtained electrode was ready for impedance measurement after carefully rinsed with 0.01 M PBS (pH 7.4) to remove non-captured cells. 

### 2.4. Cell Culture and Treatment

The human hepatocellular carcinoma cell line HepG2 was obtained from the Institute of Biochemistry and Cell Biology, Chinese Academy of Sciences (Shanghai, China). The cells were cultured in RPMI 1640 cell medium that containing 10% fetal calf serum (Gibco, Shanghai, China), penicillin (100 μg mL^−1^) and streptomycin (100 μg mL^−1^) at 37 °C. After culturing in a humidified atmosphere containing 5% CO_2_ and 95% air for 48 h, the cells in the exponential growth phase were collected and separated from the medium by centrifugation at 1200 rpm for 5 min, and then washed twice with a sterile PBS (pH 7.4). The sediment was resuspended in PBS to obtain a homogeneous solution. The cell number was determined by a cell counting instrument.

## 3. Results and Discussion

### 3.1. EIS Characterization of the Modified Electrode

**Scheme 1:**
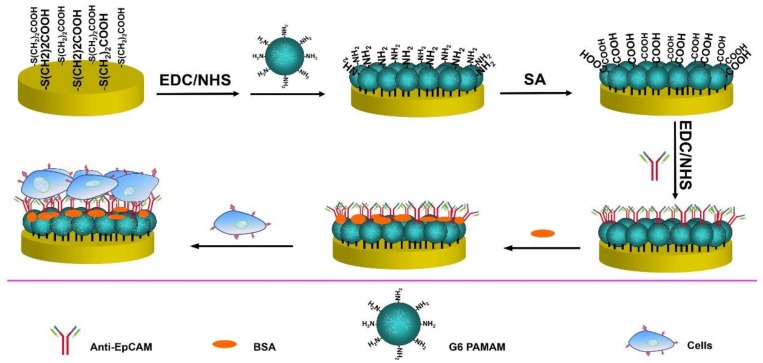
Schematic illustration of the layer-by-layer assembly process for electrochemical immunosensor construction and cancer cells detection.

**Figure 1:**
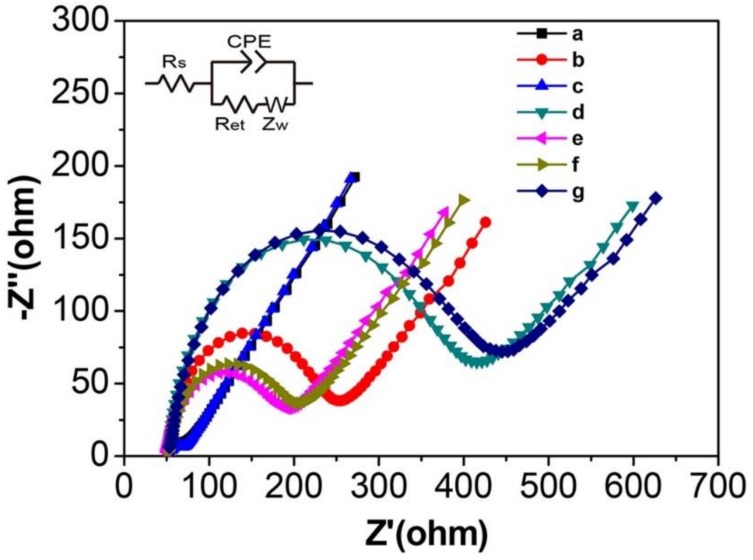
The Nyquist impedance spectra of the gold electrode modified at different stages in the presence of 0.01M pH 7.4 PBS containing 5 mM [Fe(CN)_6_]^4−/3−^ and 0.1 M KCl. (**a**) The bare gold electrode; (**b**) MPA/gold electrode; (**c**) G6 PAMAM/MPA/gold electrode; (**d**) G6 PAMAM-COOH/MPA/gold electrode; (**e**) Anti-EpCAM/G6 PAMAM-COOH/MPA/gold electrode; (f) BSA/Anti-EpCAM/G6 PAMAM-COOH/MPA/gold electrode; (**g**) Cells/BSA/Anti-EpCAM/G6 PAMAM-COOH/MPA/gold electrode. AC potential, 0.23 V; Frequency range, 100 kHz to 0.1 Hz; Voltage amplitude, 5 mV. Inset is the equivalent circuit model used to fit the impedance data.

The process for the fabrication of the electrochemical biosensor is shown in Scheme 1. For electrochemical impedance measurements, the impedance properties on the electrode surface can be obtained by an equivalent circuit model (see inset in Figure 1), which is able to offer various interface parameters between an electrode and its relevant electrolyte solution, including the ohmic resistance of the electrolyte solution (R_s_), Warburg impedance (Z_w_), constant phase element (CPE) and the surface electron transfer resistance (R_et_) [8]. Usually, R_et_ controls the interfacial electron-transfer rate of the redox probe between the solution and the electrode. In the Nyquist plot of impedance spectroscopy, it equals the semicircle diameter. Figure 1 exhibits the impedance spectra of 5 mM [Fe(CN)_6_] ^3−/4−^ containing 0.1 M KCl in 0.01 M pH 7.4 PBS at different fabrication process, respectively. Curve a showed the EIS of the bare Au electrode with an almost straight line was exhibited, which corresponded to a mass diffusional limiting electron-transfer process on the bare Au electrode. Owing to the fact that the carboxyls were negatively-charged in pH 7.4 PBS, the value of R_et_ increased obviously after the electrode was modified with MPA and the EIS showed a large interfacial R_et_ resistance compared with the bare Au electrode (curve b). When the G6 PAMAM was further incorporated onto the electrode surface, the R_et_ was reduced strongly since the G6 PAMAM possesses 256 primary amines, and the positive-charged surface can attract a negative redox probe (curve c). In addition, the G6 PAMAM can significantly increase the electrode surface area, thereby benefiting the subsequent layer-by-layer assembly procedures. Thus, the speed of electron transfer can be improved and leads to the R_et_ value even lower than that of the bare gold electrode [31]. 

When the PAMAM was completely carboxylated by SA, the negatively charged carboxyl groups repelled the electron transfer effect, resulting in a significant augment of the impedence (curve d). However, the assembly of Anti-EpCAM on the completely carboxylated PAMAM/MPA/gold electrode, the R_et_ (curve e) began to decrease. This could be ascribed to the fact that the isoionic point of Anti-EpCAM are between 8.0 and 9.5 [32]. It will be positively charged in pH 7.4 PBS buffer so that the electron transfer ability improved. Next, when blocking the unbinding surface sites through BSA, as expected, the R_et_ increased once again (curve f). Finally, the HepG2 cells recognition caused the penetration ability of the redox probe significantly reduced, resulting in a higher R_et_ value than ever before (curve g). These above EIS data can be used not only to verify the successful immobilization of MPA, PAMAM, Anti-EpCAM, BSA, and HepG2 cells onto the gold electrode surface, but can also be used to sensitive monitor the changes of R_et_ values caused by antigen–antibody recognition.

### 3.2. Cyclic Voltammetry Behavior of the Modified Electrode

**Figure 2:**
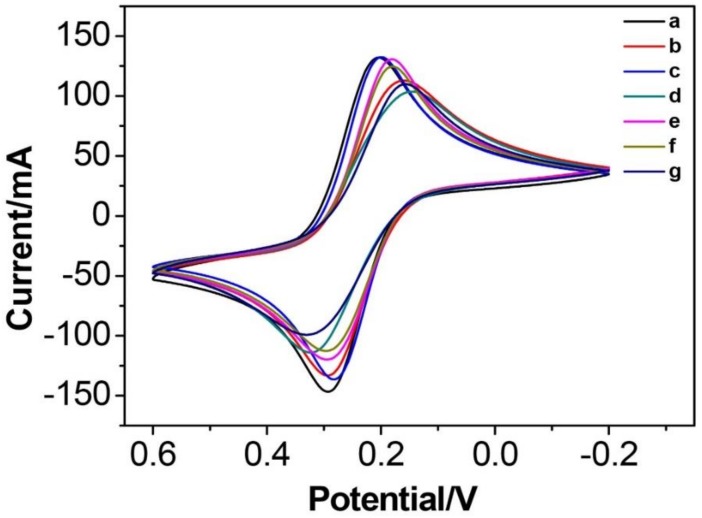
Typical CV measurements of (**a**) the bare gold electrode, (**b**) MPA/Gold electrode, (**c**) G6 PAMAM/MPA/gold electrode, (**d**) G6 PAMAM-COOH/MPA/gold electrode, (**e**) Anti-EpCAM/G6 PAMAM-COOH/MPA/gold electrode, (**f**) BSA/Anti-EpCAM/G6 PAMAM-COOH/MPA/gold electrode, and (**g**) Cells/BSA/Anti-EpCAM/G6 PAMAM-COOH/MPA/gold electrode in 0.01M pH 7.4 PBS containing 5 mM [Fe(CN)_6_]^4−/3−^ and 0.1 M KCl. Scan rate, 100 mV s^−1^; Cell concentration, 1.0 × 10^6^ cells mL^−1^.

To further validate the feasibility of the electrochemical immunosensor, the cyclic voltammetry (CV) method was adopted to analyze the modified electrode. Figure 2 shoes the CV behavior of the bare electrode for different modified electrodes in 0.01 M pH 7.4 PBS containing 5 mM [Fe(CN)_6_]^3−/4−^ and 0.1 M KCl at 100 mV/s. One can see that compared with the bare electrode (curve a), the peak current (I_p_) decreased, while the peak potential separation (ΔE_p_) increased when the surface was modified with MPA (curve b). However, the subsequent formation of PAMAM film caused the value of I_p_ increased and ΔE_p_ decreased (curve c). With the SA added to the thin film to make the amine group carboxylation, the I_p_ decreased and ΔE_p_ increased obviously (Curve d). When the Anti-EpCAM was covalently bound to the surface, the I_p_ increased and ΔE_p_ decreased (Curve e). Whereafter, the following processes of BSA modification and cell capture gave rise to the I_p_ decreased and ΔE_p_ increased consecutively. The change tendency of the CV curves indicate that the modification of MPA, carboxylation of PAMAM, absorption of BSA, and recognition of HepG2 cells can hinder the electron transfer, while assembly of PAMAM and conjugation with Anti-EpCAM can promote the electron transfer. These results are consistent well with the observed change behavior in the impedance spectroscopy (Figure 1), convincing the successful lay-by-lay assembly. 

### 3.3. Experimental Conditions Optimization 

In order to achieve the best assay performance, several important parameters including the reaction time for G6 PAMAM carboxylation, the binding time for Anti-EpCAM immobilization, and the incubation time for cancer cells recognition were investigated. 

**Figure 3:**
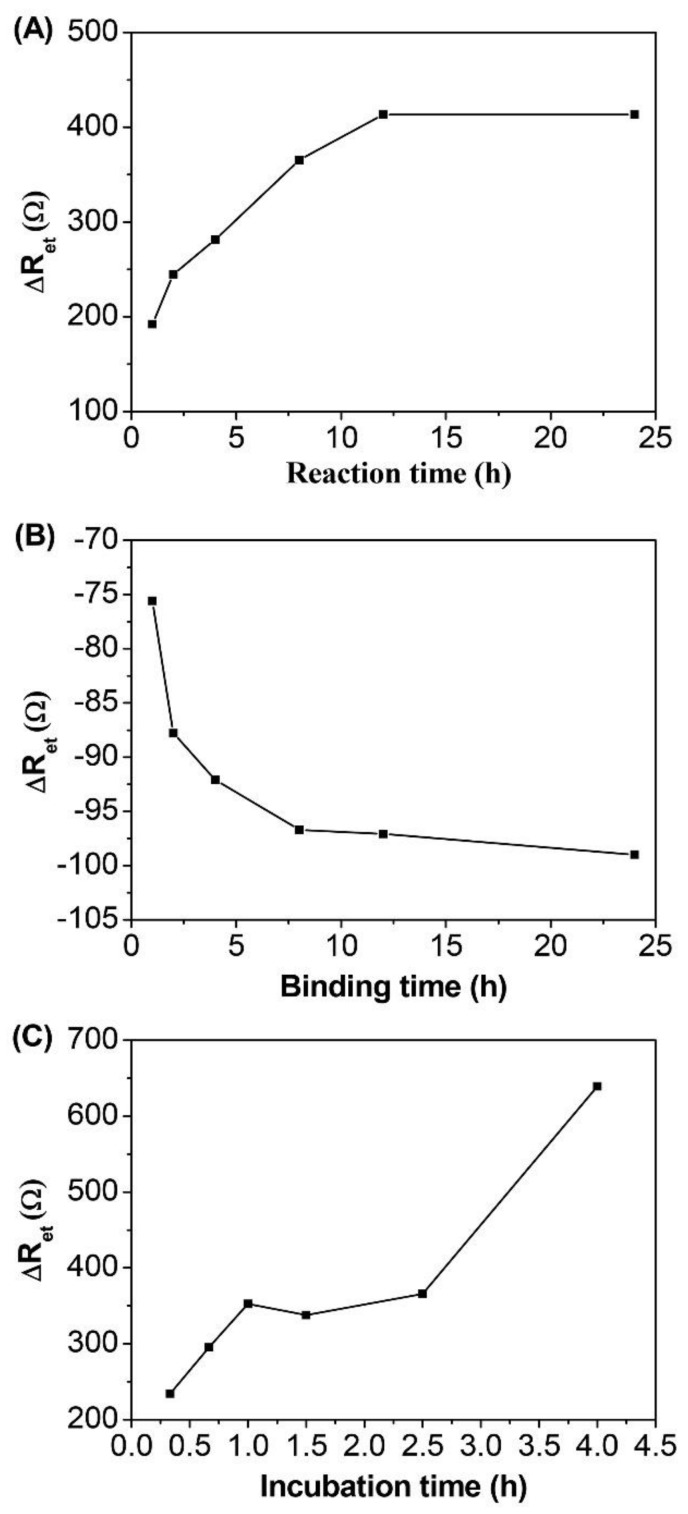
Effects of the reaction time for G6 PAMAM carboxylation (**A**), the binding time for Anti-EpCAM immobilization (**B**), and the incubation time for cancer cells recognition (**C**) on the assay performances. The ΔR_et_ in panel A is calculated as: ΔR_et_ = R_PAMAM-COOH_ − R_PAMAM_. The ΔR_et_ in panel B is calculated as: ΔR_et_ = R_Anti-EpCAM_ – R_PAMAM-COOH_. The ΔR_et_ in panel C is calculated as ΔR_et_ = R_Cells_ − R_BSA_. Cell concentration, 1.0 × 10^6^ cells mL^−1^.

It can be seen from Figure 3A that the ΔR_et_ increased gradually as the reaction time increased and reached a plateau after 12 h. Further increase of the reaction time cannot induce the ΔR_et_ to increase any more, suggesting that 12 h is sufficient for the carboxylation of all the amine groups. Therefore, we have chosen 12 h as the optimal time for G6 PAMAM carboxylation. In addition, the binding time of Anti-EpCAM to the electrode surface was tested at 4 °C to maintain the antibody activity. As shown in Figure 3B, with increasing binding time, the antibody immobilization led the ΔR_et_ values to decrease rapidly from 1 h to 8 h. Further increase of the binding time obviously cannot benefit the antibody immobilization, indicating the carboxyl groups on the surface of PAMAM are fully bound with the Anti-EpCAM molecules. Accordingly, 8 h has been selected as the best time for covalent binding. Last but not the least, the cell incubation time was optimized at 37 °C. ΔR_et_ values against the cell concentration at 1.0 × 10^6^ cells mL^−1^ at different incubation times are collected in Figure 3C. The results show that the ΔR_et_ increased slowly and then reached a turning pointing at 1 h. At the time between 1 and 2.5 h, the ΔR_et_ remained almost constant, while the ΔR_et_ shows a steep increase after 2.5 h. This means that the cells were captured on the electrode surface through a specific immune recognition reaction to achieve a saturated binding during the initial stage (0 to 1 h). The eventual increase of the ΔR_et_ after 2.5 h might be attributed to the non-specific absorption from cell lysis or apoptosis solutions owing to the fact that the cells cannot maintain a high survival rate after a long time of exposure on the electrode surface. Therefore, 1 h has been chosen for the EIS detection.

### 3.4. Specificity and Interference Study

**Figure 4:**
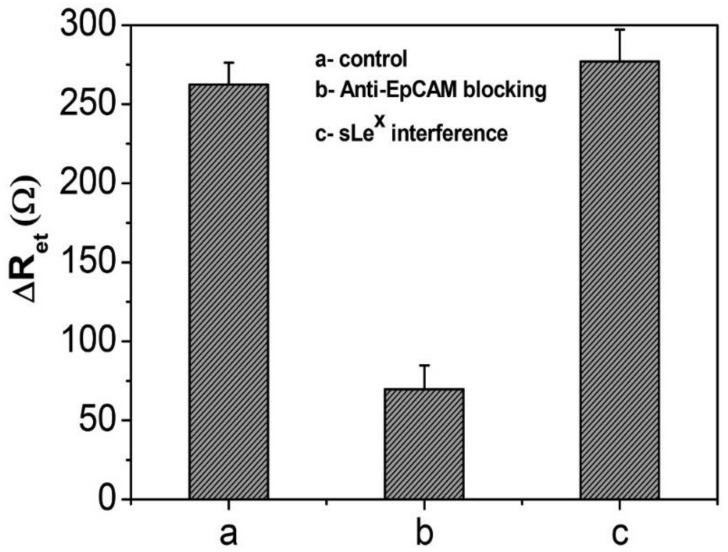
Specificity and interference investigation. Sample a is the cell suspension without any special treatments; Sample b is the cell suspension that is blocked by Anti-EpCAM. Sample c is the cell suspension added to with sLe^X^. Cell concentration: 1.0 × 10^6^ cells mL^−1^.

To affirm the interaction specificity between EpCAM and Anti-EpCAM, 1 mL 1 × 10^6^ cells mL^−1^ HepG2 were pre-incubated with 20 μL of Anti-EpCAM for 30 min in cell medium and then employed for EIS detection. Figure 4 shows the blocking effect of pre-treated HepG2 cells (sample b) only increased to a very weak impendence in contrast to the control sample (sample a). The big signal difference is reasonable since the already blocked HepG2 cells cannot bind with the Anti-EpCAM- coated electrode any more, while the free HepG2 cells still retain their binding activity, indicating the specificity for cell detection. The small increase of ΔR_et_ in sample b might be attributed to the inevitable weak physical absorption.

sLe^X^ is a tetrasaccharide that serves as a ligand for a set of cell adhesion proteins [33]. The aberrant expression of sLe^X^ on the cell surface is also related with cancer cell formation and metastasis [34], which may interfere with the measurement. We therefore prepared an interference sample (sample c) by adding 5 uL of 2 mg L^−1^ sLe^X^ into 1 mL 1 × 10^6^ cells mL^−1^ cell suspended solutions and stirred them homogeneously. Compared with the control group without sLe^X^ (sample a), the ΔR_et_ value only increased 5.55%, suggesting a negligible cross interference to the Anti-EpCAM covered electrode and an acceptable anti-interference ability for cell determination.

### 3.5. EIS Detection of HepG2 Cells

**Figure 5:**
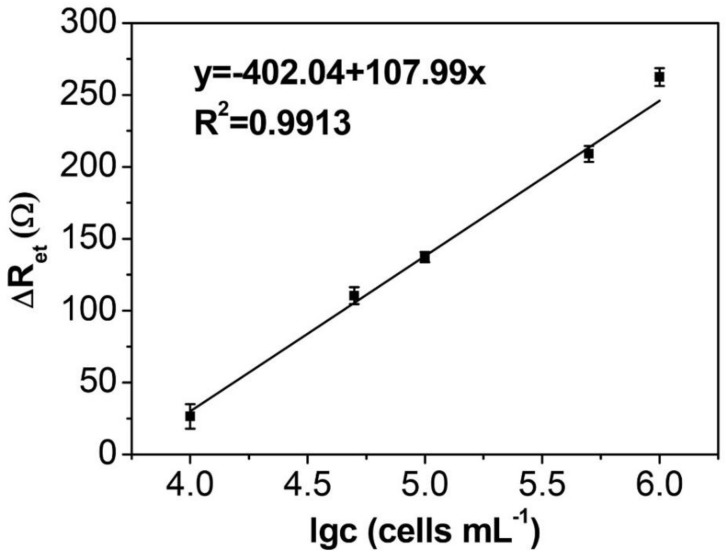
Plot of the ΔR_et_ (ΔR_et_ = R_cells_ − R_BSA_) against the target cancer cell at different concentrations. The data are obtained from at least three parallel tests.

Under optimal conditions, the EIS measurements of the [Fe(CN)_6_]^3−/4−^ probe before and after the incubation of HepG2 cells at various concentrations were performed and the results are gathered in Figure 5. As presented, the ΔR_et_ value exhibited a linear response with the logarithm of HepG2 cell concentrations ranging from 1.0 × 10^4^ to 1.0 × 10^6^ cells mL^−1^. The linear regression equation was deduced as Y = −402.04 + 107.99X (R^2^ = 0.9913), and the detection limit was estimated to be 2.1 × 10^3^ cells mL^−1^ according to the 3σ rule, which was comparable to previously reported values for cell detection sensors [18,35,36]. The relative standard deviation (RSD) of the detection results were all lower than 6%, showing acceptable reproducibility of the fabricated biosensor. Of note, we must point out that the sensitivity is still not powerful enough, and should be further enhanced to meet the application demands. However, in this study, our goal was to build a new electrochemical analytical tool for cell detection on the basis of a PAMAM sensing platform. We, therefore, expect this new method will pave a new way and encourage subsequent studies in the field of biomedical analysis.

## 4. Conclusions

In summary, by using G6 PAMAM as the assembly component, we have developed a label-free electrochemical immunosensor for cancer cell detection based on the recognition between Anti-EpCAM and EpCAM on a modified electrode surface. Without any complex operations or tedious labeling, the cell capture resulted in a significant increase of the impedance value for EIS measurements using [Fe(CN)_6_]^3−/4−^ as the electrochemical probe. The ΔR_et_ was linearly proportional to the HepG2 cell concentration in a wide linear range associated with a good sensitivity and specificity. We expect that the proposed detection platform will lead to a potential analytical tool for biochemical analysis.
